# Journal of Foot and Ankle Research: the first ten years

**DOI:** 10.1186/s13047-018-0287-9

**Published:** 2018-08-01

**Authors:** Hylton B. Menz, Alan M. Borthwick, Catherine J. Bowen

**Affiliations:** 1 School of Allied Health, College of Science, Health and Engineering, La Trobe University, Melbourne, VIC Australia; 2 Faculty of Health Sciences, University of Southampton, Southampton, UK

## Abstract

Journal of Foot and Ankle Research (JFAR) was launched in July 2008 as the official research publication of the Society of Chiropodists and Podiatrists (UK) and the Australasian Podiatry Council, replacing both the British Journal of Podiatry and the Australasian Journal of Podiatric Medicine. This editorial celebrates the 10 year anniversary of the journal.

## Introduction

*Journal of Foot and Ankle Research (JFAR)* was launched in July 2008 as the official research publication of the Society of Chiropodists and Podiatrists (UK) and the Australasian Podiatry Council, replacing both the *British Journal of Podiatry* and the *Australasian Journal of Podiatric Medicine* [1]. *JFAR* was developed to meet the growing need for an international platform for the publication of research within the podiatry profession [2–6], and the timing of its launch coincided with the rise of open access publishing – an innovative publication model which enables free full-text access to anyone with an internet connection [7]. Since its inception, *JFAR* has been published by BioMed Central (now BMC), one of the pioneers of scholarly open access. This editorial celebrates the journal’s 10-year anniversary by summarising the key achievements of the journal between 2008 and 2018.

## Publication characteristics

**Fig. 1:**
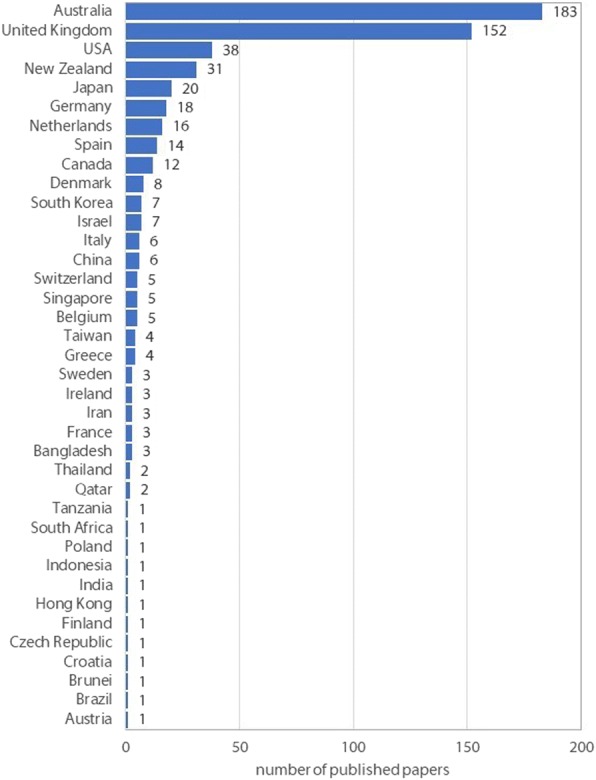
Country of corresponding author of *JFAR* papers, 2008–2018

Since July 2008, 442 papers have been published in *JFAR*, with authors from 38 different countries (see Fig. 1). *JFAR* has also published 14 conference proceedings, including the biennial Australasian Podiatry Conference (2011, 2013, 2015 and 2017), the annual College of Podiatry conference (2010, 2013, 2014, 2015, 2016 and 2017), and the International Foot and Ankle Biomechanics Community (i-FAB) conference (2008, 2012 and 2014), and five article collections: the Diabetic Foot (2012), the Rheumatoid Foot (2013), the Paediatric Foot (2015) and the Science and Sociology of Footwear (2018).

**Fig. 2:**
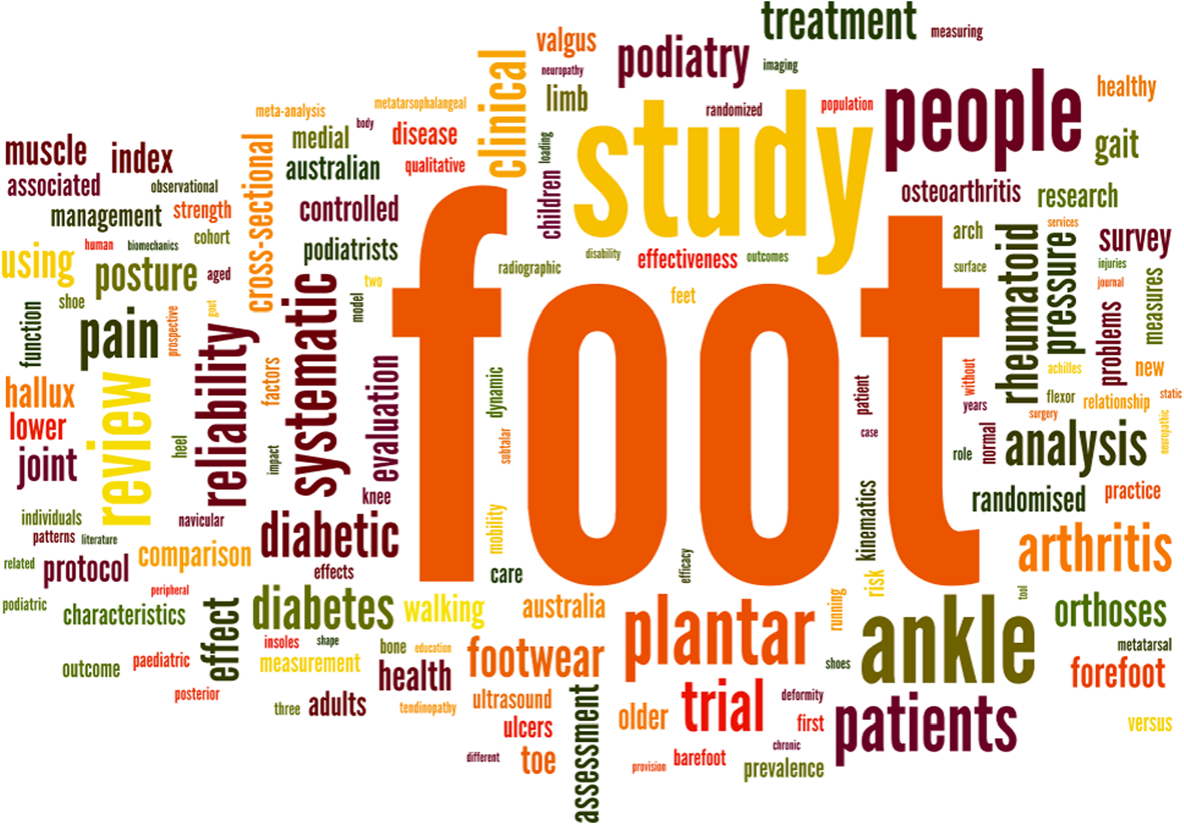
Word cloud of titles of papers published in *JFAR*, 2008–2018

According to the Scopus® database, the ten most common keywords used in the abstracts of *JFAR* papers (excluding humans, male and female) were foot, physiology, podiatry, gait, ankle, diabetes, shoes, biomechanics, foot orthoses and footwear. A word cloud representing the 150 most common words used in the titles of all 442 papers published in the journal is shown in Fig. 2.

## Peer review metrics

*JFAR* receives between 100 and 150 papers each year, of which approximately 50 are accepted for publication. The acceptance rate is trending downwards and is currently 36%. The average time to a first editorial decision for reviewed manuscripts is 55 days, and the average time from submission to acceptance is 112 days (this includes the time taken to find peer reviewers and the time taken for authors to revise their manuscripts). Over the past 10 years, the time taken to find peer reviewers has increased. This reflects the growing global burden of peer review in the biomedical literature, which has been estimated at 63 million hours per year [8].

## Fate of rejected papers

To determine the fate of papers rejected from *JFAR*, we extracted the first 100 rejected papers from the editorial database, and searched PubMed and Google Scholar in April 2018 using the title, key words and author names of each paper. Of these papers, 39 could not be located in another journal. The remaining 61 papers were subsequently published in 45 different journals (including six foot and ankle journals), the most common destination journals being *The Foot* (seven papers) and *Journal of the American Podiatric Medical Association* (six papers). The time period between rejection from *JFAR* and subsequent publication in another journal ranged from 0 to 58 months (median 16). These data provide evidence of a journal hierarchy amongst foot and ankle researchers, with papers eventually published in *The Foot*, *Journal of the American Podiatric Medical Association*, *Diabetic Foot and Ankle*, *Foot and Ankle Online Journal*, *Foot and Ankle Specialist* and *Foot and Ankle Surgery* first being submitted to *JFAR*. However, it is also likely that *JFAR* receives manuscripts rejected from other journals, particularly specialist biomechanics, sports medicine, orthopaedics, diabetes and rheumatology journals.

## Journal performance metrics

**Fig. 3:**
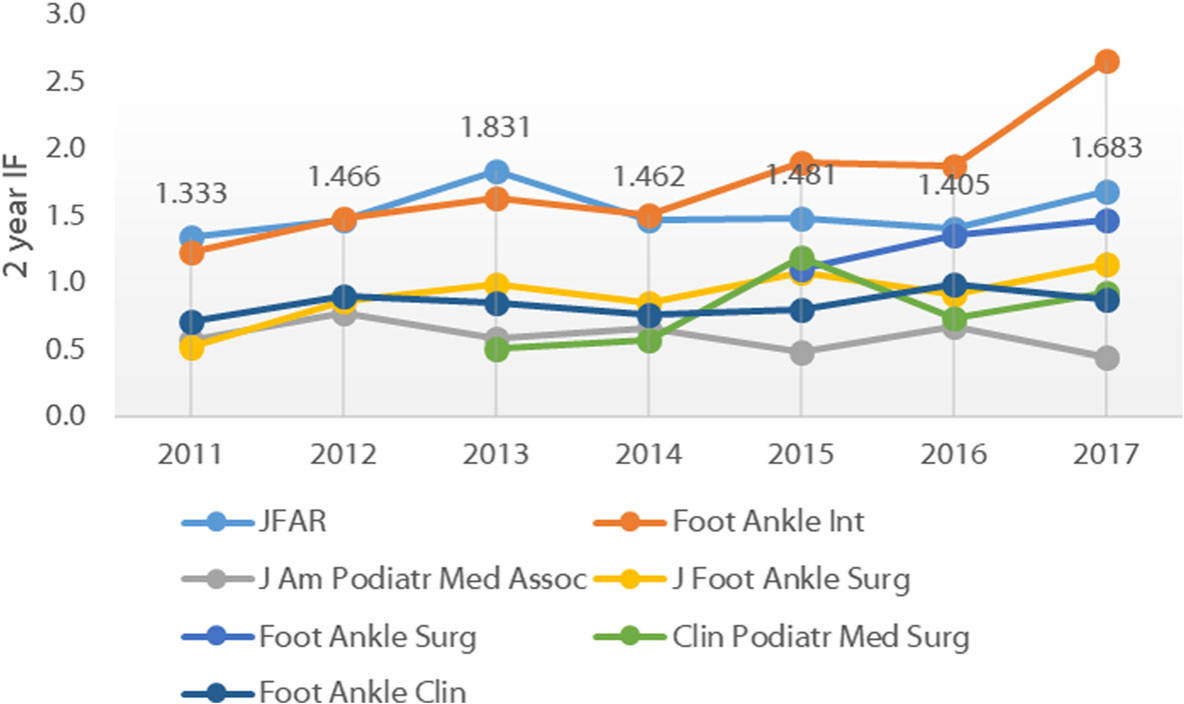
Two-year Impact Factors for foot and ankle journals, 2011–2016

**Fig. 4:**
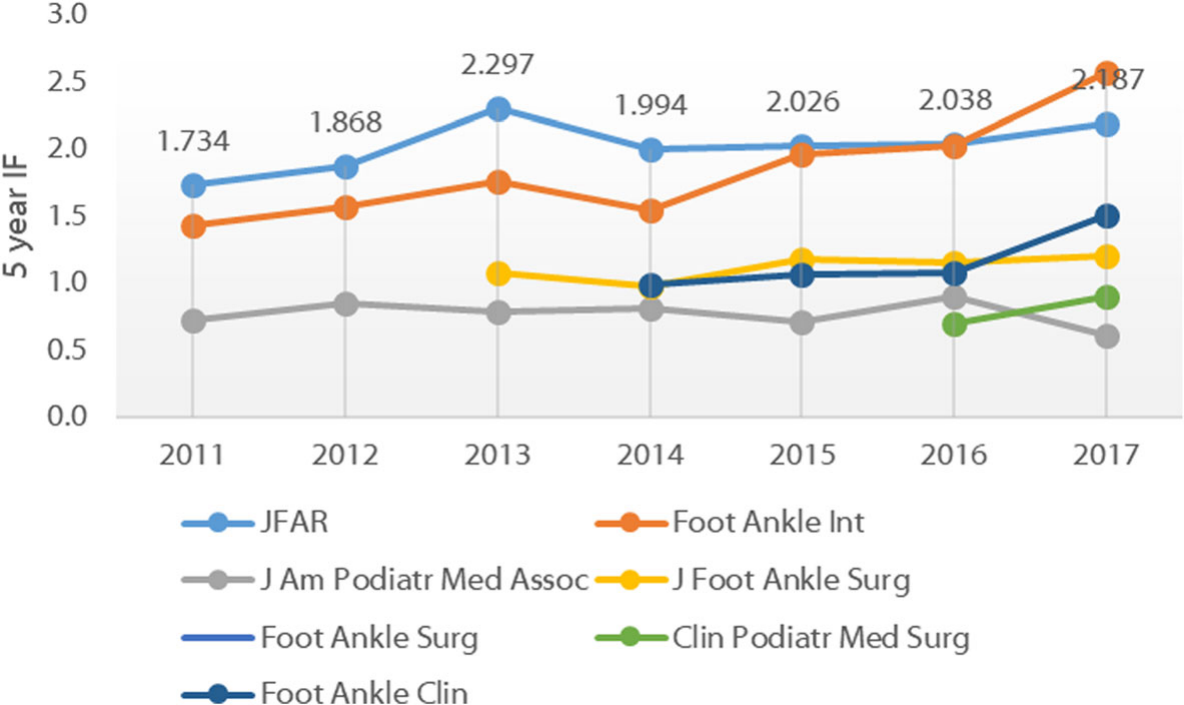
Five-year Impact Factors for foot and ankle journals, 2011–2016

There are several citation-based metrics to evaluate journal performance. By far the most widely used is the Impact Factor (IF), first developed in 1955 [9]. The IF represents the average number of citations received per paper published in that journal during the two preceding years (2-year IF) or five preceding years (5-year IF). *JFAR* was formally accepted for IF tracking by Thomson Reuters (now Clarivate Analytics) in November 2011, and received its first IF (1.333) in 2012 [10]. Since this time, the IF has fluctuated (largely as a function of the total number of papers published per year), with the most recent IFs available indicating that *JFAR* has the 2nd highest 2-year IF (1.683) and 5-year IF (2.187), behind *Foot and Ankle International*. The 2-year and 5-year IFs for all foot and ankle journals are shown in Figs. 3 and 4, respectively.

**Fig. 5:**
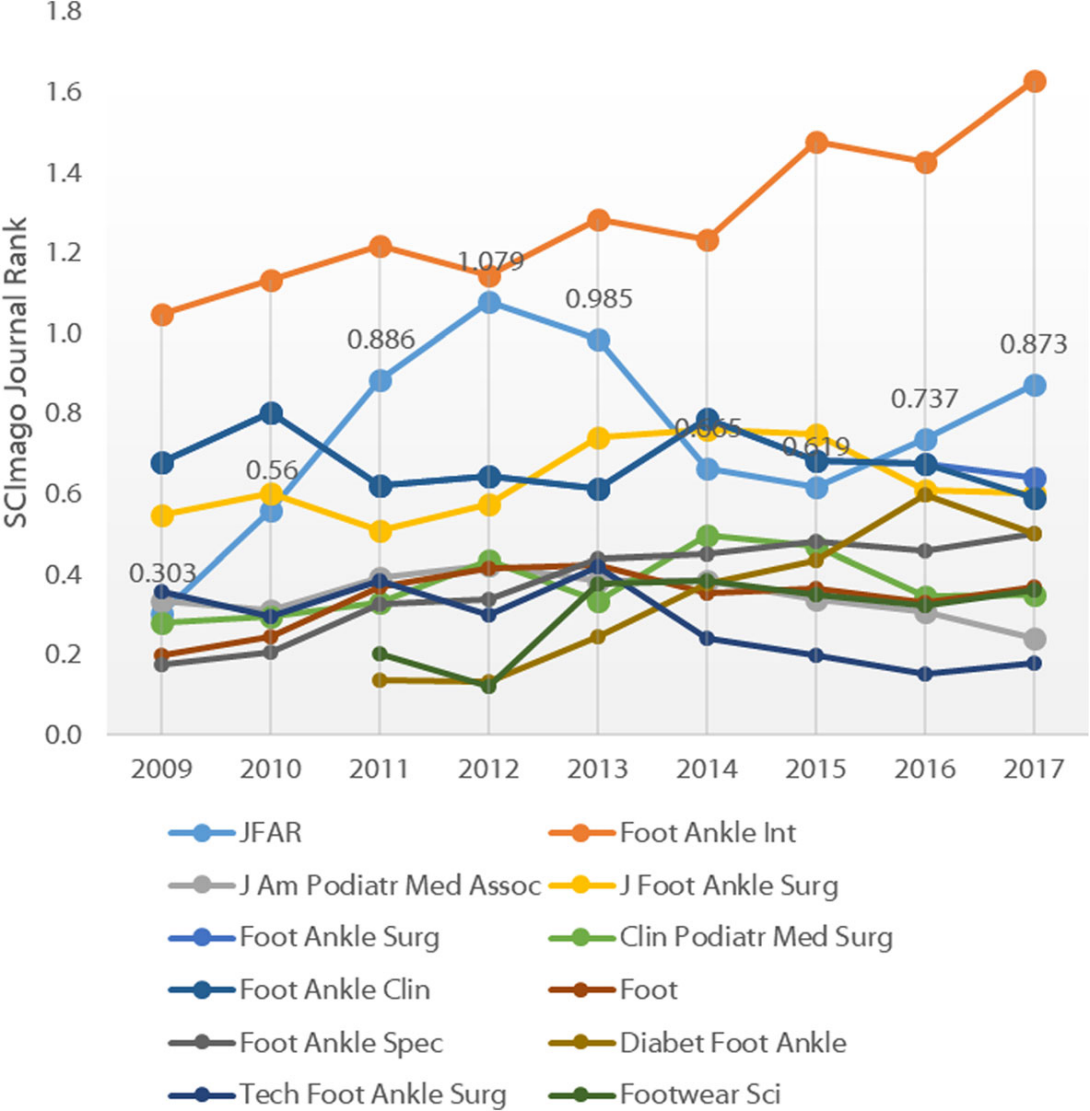
SCImago Journal Rank for foot and ankle journals, 2009–2017

**Fig. 6:**
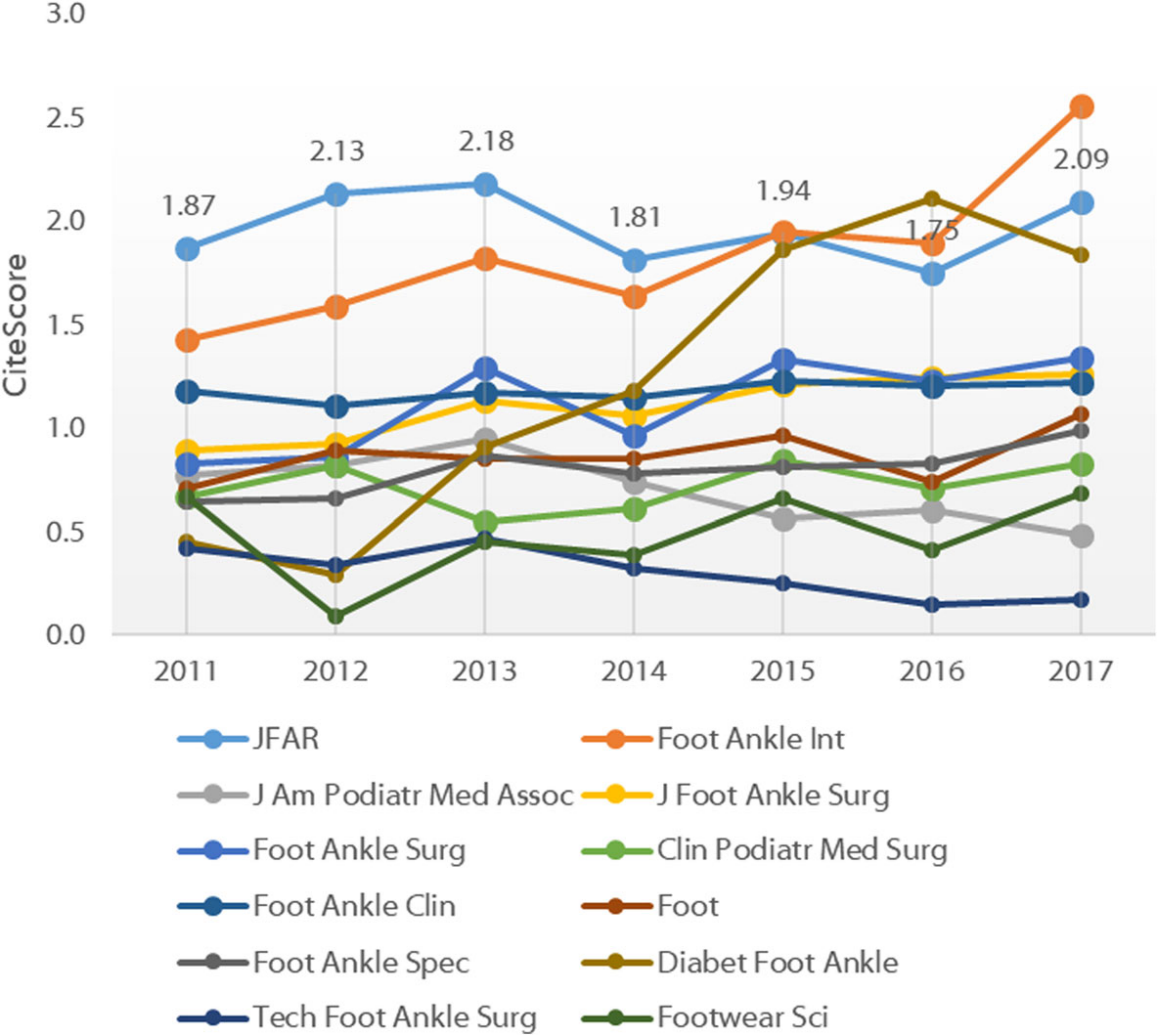
CiteScore for foot and ankle journals, 2011–2017

More recently, the SCImago Journal Rank (SJR) has been developed by the technology company SCImago Lab [11]. The SJR uses Elsevier’s more extensive Scopus® database and uses a more complex algorithm similar to Google’s PageRank which accounts for both the number of citations and the prestige of the journals where the citations came from. *JFAR*’s SJR (0.873) is second only to *Foot and Ankle International* (Fig. 5). Finally, Elsevier’s new CiteScore metric [12], which reflects the average citations per document that a title receives over a three-year period and incorporates all document types, shows *JFAR* (CiteScore 2.09) ranked in 2nd position behind *Foot and Ankle International* (Fig. 6).

These data clearly show that *JFAR* has performed extremely well for a relatively young journal. However, *JFAR*’s position on citation metrics, as outlined in our 2012 editorial, remains that “rather than agonising over ubiquitous yet flawed journal performance metrics, we will continue to make editorial decisions based on the relevance and scientific quality of individual manuscripts” [10].

## Notable papers

The impact of individual papers can be assessed in several ways: the number of accesses, the number of citations, and the number of mentions on social media. Table 1 shows the top ten papers according to each of these metrics, using data from the *JFAR* website, the Scopus® database, and Altmetric Attention Scores, respectively. The most accessed manuscript in the 10-year history of the journal is Bristow’s clinical guideline for the recognition of malignant melanoma [13], the most cited paper is Redmond et al’s normative values for the Foot Posture Index [14], and the paper with the most social media coverage is Neal et al’s systematic review of foot posture as a risk factor for lower limb overuse injury [15].

**Table 1:** Top ten papers published in *JFAR* between 2008 and 2018 according to accesses, citations and Altmetric scores

Accesses (source: *JFAR* website)^a^	Reference	Score
Clinical guidelines for the recognition of melanoma of the foot and nail unit	[13]	46,459
A consensus definition and rating scale for minimalist shoes	[27]	7181
Patterns of foot complaints in systemic lupus erythematosus: a cross sectional survey	[28]	6478
Physical therapies for Achilles tendinopathy: systematic review and meta-analysis	[29]	6035
Plantar calcaneal spurs in older people: longitudinal traction or vertical compression?	[30]	5971
Gait and Lower Limb Observation of Paediatrics (GALLOP): development of a consensus based paediatric podiatry and physiotherapy standardised recording proforma	[31]	5766
Normative values for the Foot Posture Index	[14]	5436
Diabetic foot: prevalence, knowledge, and foot self-care practices among diabetic patients in Dar es Salaam, Tanzania – a cross-sectional study	[32]	5311
Non-surgical treatment of hallux valgus: a current practice survey of Australian podiatrists	[33]	5092
Challenging the foundations of the clinical model of foot function: further evidence that the Root model assessments fail to appropriately classify foot function	[34]	4677
Citations (source: Scopus®)		
Normative values for the Foot Posture Index	[14]	152
Prevalence of hallux valgus in the general population: A systematic review and meta-analysis	[35]	140
Prevalence and correlates of foot pain in a population-based study: The North West Adelaide health study	[36]	126
A protocol for classifying normal- and flat-arched foot posture for research studies using clinical and radiographic measurements	[37]	90
Diagnostic imaging for chronic plantar heel pain: A systematic review and meta-analysis	[38]	81
Reliability of the TekScan MatScan® system for the measurement of plantar forces and pressures during barefoot level walking in healthy adults	[39]	63
Foot posture influences the electromyographic activity of selected lower limb muscles during gait	[40]	61
Physical therapies for Achilles tendinopathy: systematic review and meta-analysis	[29]	55
Development and evaluation of a tool for the assessment of footwear characteristics	[41]	54
Reliability and normative values for the foot mobility magnitude: A composite measure of vertical and medial-lateral mobility of the midfoot	[42]	51
Social media impact (source: Altmetric Attention Score)		
Foot posture as a risk factor for lower limb overuse injury: a systematic review and meta-analysis	[15]	132
A consensus definition and rating scale for minimalist shoes	[27]	85
Challenging the foundations of the clinical model of foot function: further evidence that the Root model assessments fail to appropriately classify foot function	[34]	70
The effect of high-top and low-top shoes on ankle inversion kinematics and muscle activation in landing on a tilted surface	[43]	56
Clinical guidelines for the recognition of melanoma of the foot and nail unit	[13]	51
Higher frequency of hamstring injuries in elite track and field athletes who had a previous injury to the ankle - a 17 years observational cohort study	[44]	44
Effect of thong style flip-flops on children’s barefoot walking and jogging kinematics	[45]	38
The effect of foot orthoses and in-shoe wedges during cycling: a systematic review	[46]	36
The typically developing paediatric foot: how flat should it be? A systematic review	[47]	36
The Foot Orthoses versus Hip eXercises (FOHX) trial for patellofemoral pain: a protocol for a randomized clinical trial to determine if foot mobility is associated with better outcomes from foot orthoses	[48]	36

^a^Data for 2016 onwards only. As there have been a number of platform changes over the past 10 years, it is not possible to accurately calculate ‘all time’ accesses

Another way to assess the relative importance of papers is by applying the hierarchy of evidence, which places systematic reviews above all other study designs, including randomised trials, non-randomised studies, observational studies, case series studies and case reports. In this context, it is pleasing to note that *JFAR* has published a total of 31 systematic reviews. These reviews have summarised the best available evidence for a wide range of topic areas, including the effectiveness of treatments such as foot orthoses [16], stretching [17], dry needling [18], laser therapy [19], prolotherapy [20], extracorporeal shock-wave therapy [21] and soft tissue surgery [22]. Systematic reviews are an extremely valuable resource for clinicians trying to keep up to date with the growing body of research literature pertaining to the treatment of foot disorders.

## Website traffic

**Fig. 7:**
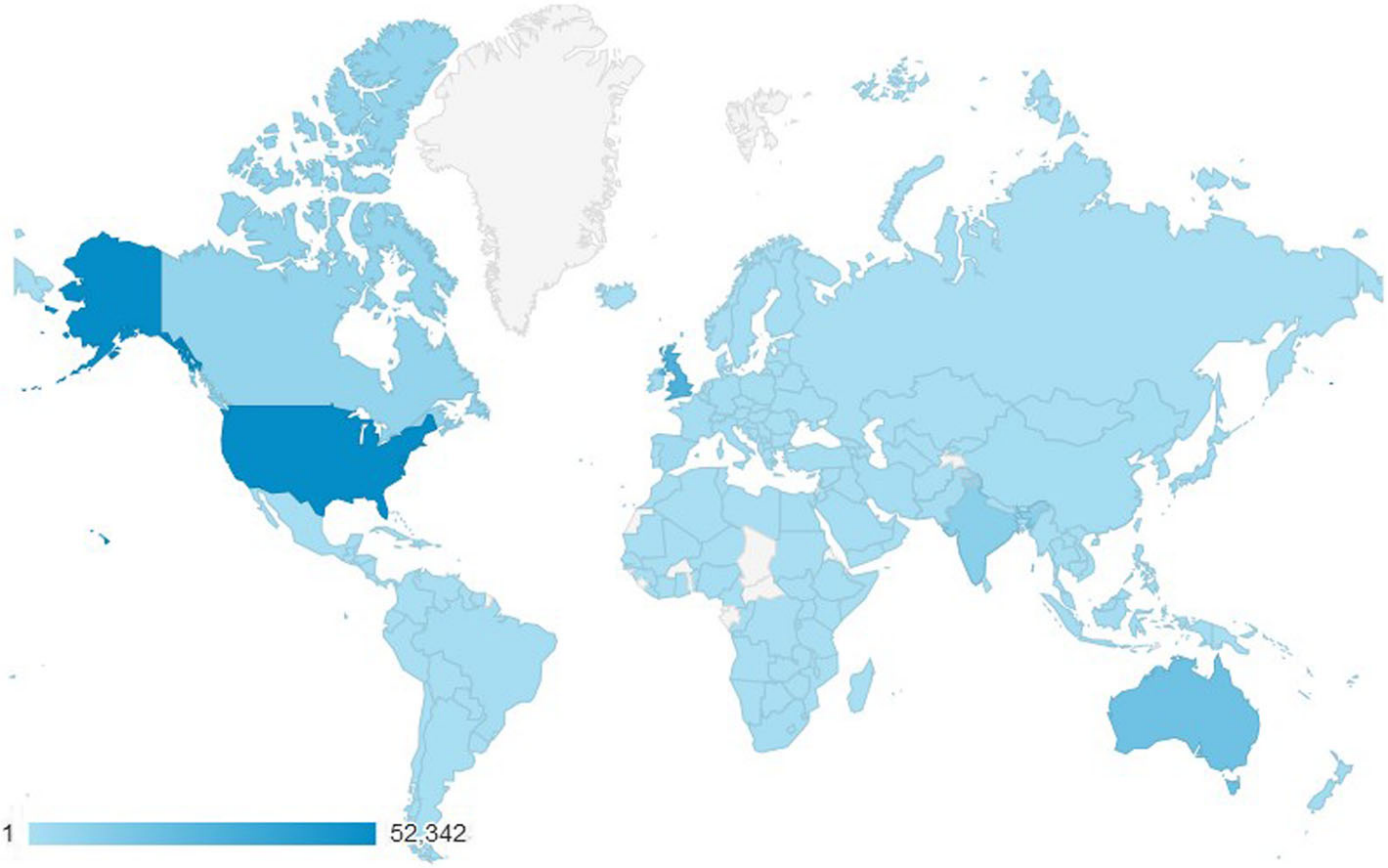
*JFAR* website accesses by country, 2017. Source: Google Analytics

The BMC website attracts over 20 million visits each month. In 2017, *JFAR*’s dedicated site received 264,565 page views, with an average of 19,214 views per month. The website was accessed by readers from most countries in the world, with the highest number of accesses from the USA (59,971), followed by the UK (43,733), Australia (31,260), India (12,838) and Canada (8218). See Fig. 7.

## Future directions

As the first foot and ankle journal to fully embrace open access publishing, *JFAR* has been an early adopter of innovations in academic publishing. Consistent with BMC’s ethos of transparency, we operate an open peer review process (where authors’ and reviewers’ identities are disclosed), and we publish all peer reviews on our website. The BMC platform also allows for non-traditional content to be uploaded to support manuscripts, including video files [23] and downloadable 3-dimensional models [24].

Engaging readers, however, particularly time-poor clinicians, is an ongoing challenge for all scholarly journals. Relatively recent innovations to improve readability, engagement and translation include video abstracts [25] and infographics – brief summaries of research papers that use data visualisation techniques to convey key messages [26]. Several journals have trialed infographics, either to supplement full papers or as stand-alone, peer-reviewed publications. At *JFAR*, we will explore all strategies for improving the reader experience while ensuring that the information provided is as accurate and unbiased as possible.

## Editorial changes

Professor Hylton Menz (Editor-in-Chief, Australia) and Professor Alan Borthwick, OBE (Editor-in-Chief, UK) will step down from their roles at the end of July 2018. The new Editors-in-Chief will be Professor Keith Rome (AUT University, Auckland, New Zealand) and Professor Catherine Bowen (University of Southampton, UK), the new Deputy Editors will be Dr. Andrew Buldt (La Trobe University, Australia) and Dr. Michelle Spruce (Blandford Forum and Wareham, UK) and the Associate Editors will be Mr Daniel Bonanno (La Trobe University, Australia), Dr Cylie Williams (Monash University, Australia) and Dr Anita Williams (Salford University, UK).

## Thanks and acknowledgements

The authors would like to sincerely thank the following individuals for contributing to *JFAR*’s success over the past 10 years: past and present editorial colleagues (Mr Mike Potter, Associate Professor Karl Landorf, Associate Professor Shannon Munteanu, Dr. Farina Hashmi, Professor Joshua Burns, Professor Keith Rome, Dr. Anita Williams, Mr. Daniel Bonanno and Dr. Andrew Buldt), past and present BMC journal development managers (Shivani Gore, Miranda Wilson-Woods and Victoria Slim) and past and present Board of Management members (John Price, Alison Petchell, Graham Ramsey, Dr. Sue Whicker, Kelli Cheales, Dr. Anita Raspovic, Damien Mitsch, George Wilson and Nello Marino from the Australian Podiatry Association, Joanna Brown, Rosemary Gillespie, Clare Richards and Paul Chadwick from the College of Podiatry/Society of Chiropodists and Podiatrists, and Stephen Hartman from the Canadian Federation of Podiatric Medicine). We would also like to thank our editorial board and peer reviewers for their tireless efforts in assessing the suitability of papers for publication.

## Abbreviations

Clin Podiatr Med Assoc: Clinics in Podiatric Medicine and Surgery
Diabet Foot Ankle: Diabetic Foot and Ankle
Foot Ankle Clin: Foot and Ankle Clinics
Foot Ankle Int: Foot and Ankle International
Foot Ankle Spec: Foot and Ankle Specialist
Foot Ankle Surg: Foot and Ankle Surgery
Footwear Sci: Footwear Science
IF: Impact factor
J Am Podiatr Med Assoc: Journal of the American Podiatric Medical Association
J Foot Ankle Surg: Journal of Foot and Ankle Surgery
JFAR: Journal of Foot and Ankle Research
Tech Foot Ankle Surg: Techniques in Foot and Ankle Surgery
